# Acoustic Features in ALS: Taking a Pause to Appreciate a Novel Remote Respiratory Monitoring Strategy

**DOI:** 10.1002/mus.70056

**Published:** 2025-11-06

**Authors:** Aaron S. Zelikovich, Jason Ackrivo

**Affiliations:** 1 Department of Neurology, Perelman School of Medicine, University of Pennsylvania, Philadelphia, Pennsylvania, USA; 2 Pulmonary, Allergy, and Critical Care Division, Department of Medicine, Perelman School of Medicine at the University of Pennsylvania, Philadelphia, Pennsylvania, USA

**Keywords:** amyotrophic lateral sclerosis, respiratory failure, respiratory function, speech impairment, telemedicine

Over the past decade, people with amyotrophic lateral sclerosis (PALS) have experienced a renaissance of clinical trial opportunities, such as the multiple drug trials through the HEALEY Platform Trial [1]. The COVID-19 pandemic accelerated the need for telemonitoring throughout medicine. In response, ALS clinics that normally provide in-person multidisciplinary care with quarterly respiratory testing, physical therapy assessments, and neurological exams found it necessary to develop remote mechanisms of care for PALS [2]. The rapid advancement of telemedicine has benefitted PALS by improving access to care while reducing travel burden. Consequently, ALS researchers have sought novel remote assessment tools to monitor PALS in their home environment [3].

The recent publication, “A Preliminary Investigation of Acoustic Features for Remote Monitoring of Respiration in ALS” by Connaghan et al., details a novel approach to remote assessment of respiratory function in PALS using voice recordings to analyze speech pattern changes in pause time, total pause duration, pause events, speaking rate, and articulation rate [4]. The study describes a single center experience in PALS who underwent standardized respiratory testing in person followed by a remote speech assessment. Participants read aloud a standard one-paragraph *Bamboo Passage* that was spoken and recorded on a smartphone through the Beiwe application created to analyze speech patterns. The audio was analyzed in comparison to the patient’s vital capacity and self-reported Amyotrophic Lateral Sclerosis Functional Rating Scale- Revised (ALSFRS-R) scores that were collected within a 16-day window of each other.

The authors analyzed multiple acoustic features and found features related to pauses in speech to be the most relevant. The pause time, total pause duration, average pause duration, and number of pause events were associated with vital capacity but only pause time showed a statistically significant correlation with the ALSFRS-R scores. Pause time had the strongest correlation with vital capacity (*r* = −0.62), suggesting a moderate inverse correlation between vital capacity and pause time. The authors reported an acceptable classification between percent pause time and self-reported ALSFRS-RSE (Revised ALS Functional Rating Scale Self-Entry version) respiratory subscore when using a cut-off score of 11 (AUC 0.78), as patients with subjective respiratory weakness had longer pause times.

Key study limitations include the sample size, reliance on vital capacity as a gold standard, and cross-sectional study design. Of the 36 participants, six had bulbar-onset ALS. Historically, bulbar involvement causes falsely low vital capacity due to the inability to obtain a full seal on the spirometry interface. The mean slow vital capacity reported was 75% of predicted with a range of 33%–116%. The large range of slow vital capacity theoretically provided a sample of patients across the spectrum of disease, from severe respiratory involvement to no respiratory involvement. This study had a small sample size, preventing subcohort analyses by education level, bulbar onset versus limb onset, or rapid versus slowly progressive disease. The cross-sectional study design precluded investigating longitudinal relationships between respiratory function and speech features.

Alternative respiratory monitoring options to conventional in-clinic respiratory assessments for ALS include home vital capacity assessments [5], noninvasive positive pressure ventilation (NIPPV) remote monitoring [2], and transcutaneous carbon dioxide (PtcCO_2_) [6, 7]. All three alternatives require specialized training, are resource-intensive, and carry additional costs, thus limiting the routine use in the home setting. The use of acoustic features in ALS as a form of remote respiratory monitoring has several advantages, including low cost, minimal patient burden, and frequent repeatability. Ideal remote monitoring should include measurements with low patient burden that are repeatable and reliable. However, the study’s cross-sectional design and lack of disease controls limit the ability to comment on the reproducibility and reliability of using acoustic features to differentiate between ALS and other diseases.

Potential advantages to a practical, repeatable remote assessment in ALS include improving the timeliness of interventions and providing a novel biomarker. NIPPV has been shown to improve survival in PALS [8]; however, the ideal time to initiate NIPPV remains unclear, often causing delays until the development of significant respiratory impairment. Low-cost remote monitoring tools, such as acoustic features, may permit an increase in the frequency of respiratory assessments between clinic visits and therefore may improve the timeliness of NIPPV initiation. With further study, it is plausible that frequent remote monitoring of acoustic features may enable triaging patients to an earlier in-person appointment for spirometry and consideration of NIPPV. Additionally, future work may determine whether ALS clinical trials could use acoustic features as a secondary outcome for respiratory function.

The study by Connaghan et al. adds to the growing literature on potential biomarkers for home monitoring of respiratory function in PALS. In future work, the authors propose studying a combination of acoustic pause time metrics longitudinally. With further study, speech analysis may become a clinically meaningful tool to provide an easy and cost-effective way for telemonitoring of respiratory function in PALS in both clinical care and research. We applaud the authors for their innovative approach, and we look forward to future studies that clarify the best practices of this exciting new biomarker for respiratory function in ALS.

## Data Availability

Data sharing not applicable to this article as no datasets were generated or analysed during the current study.
